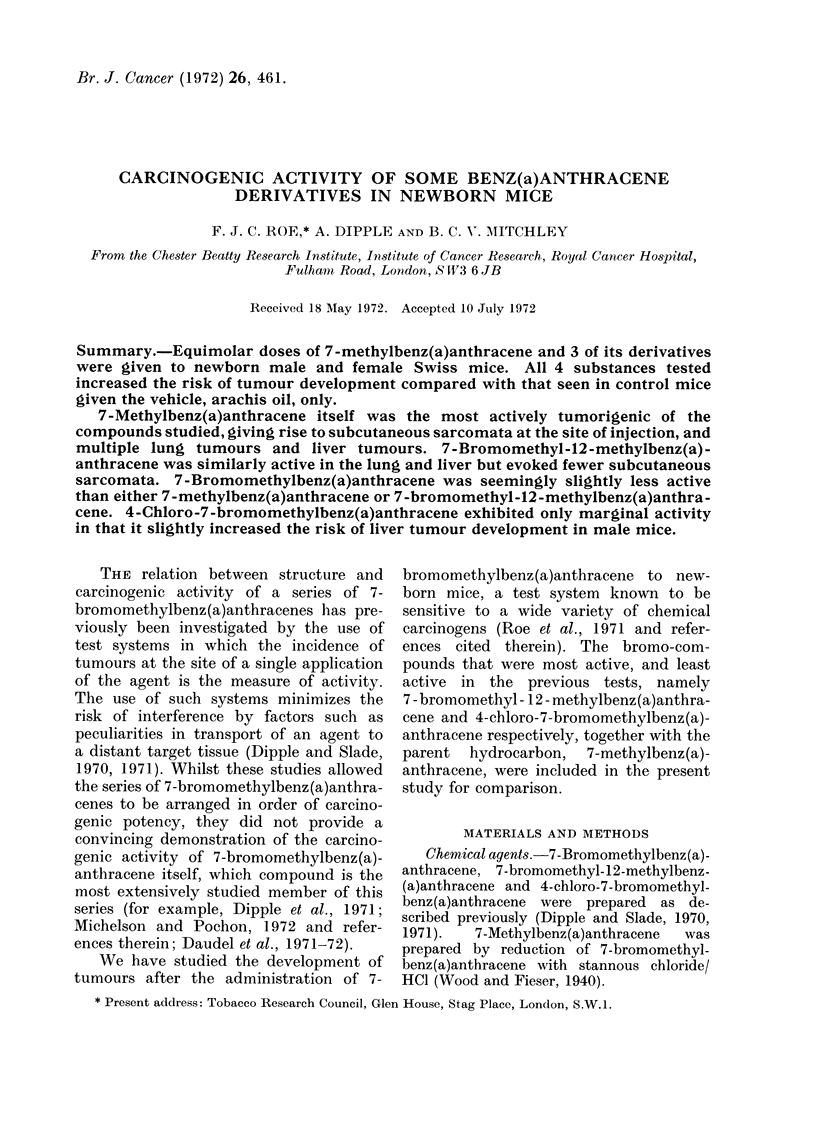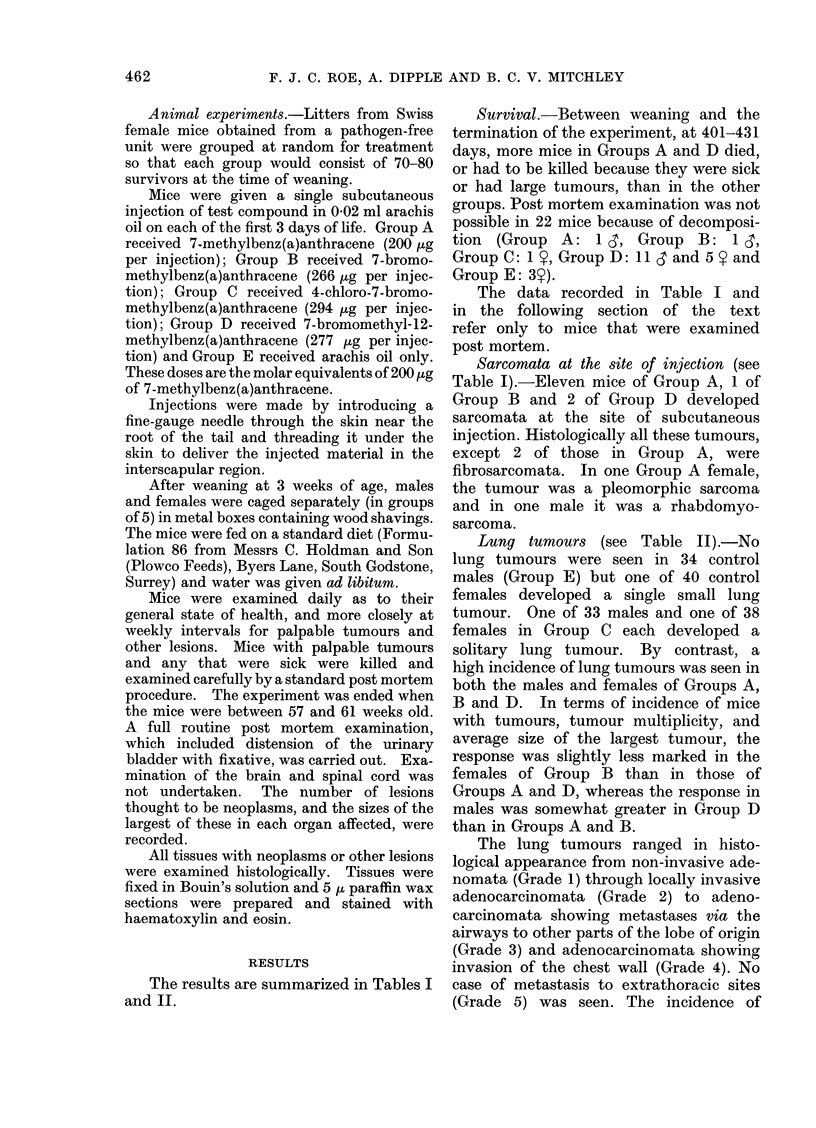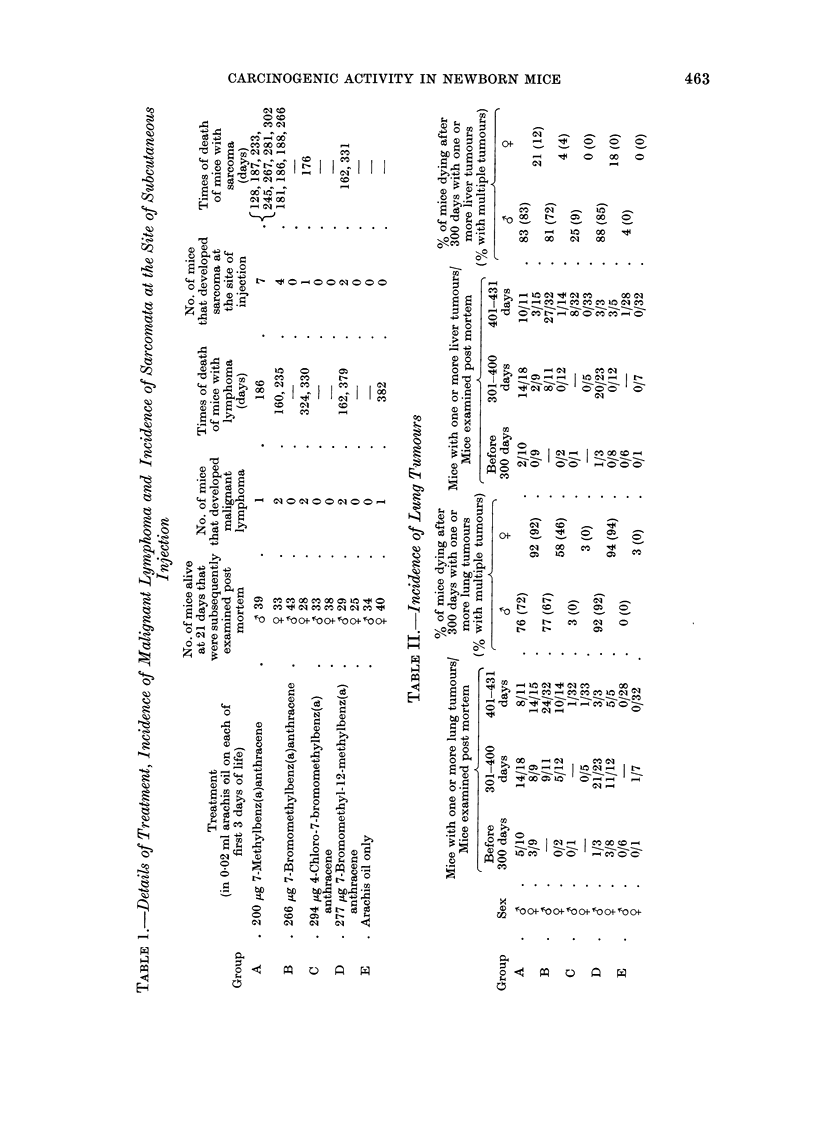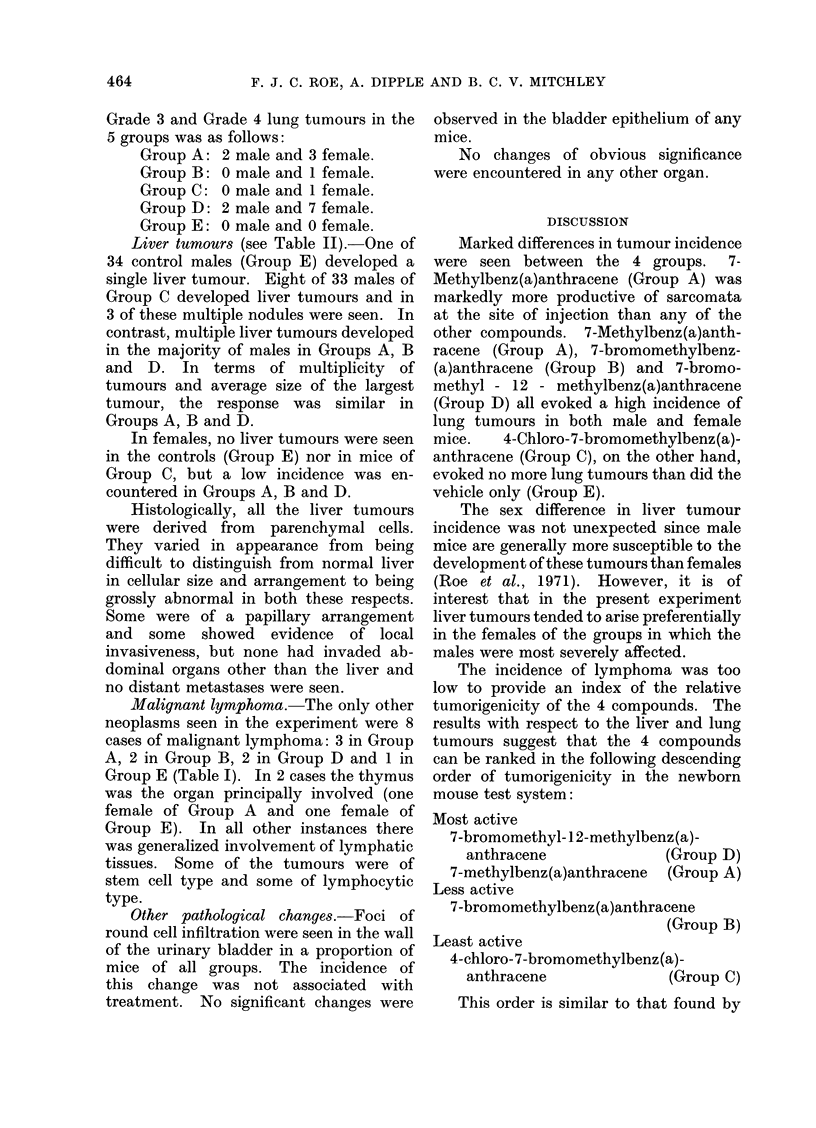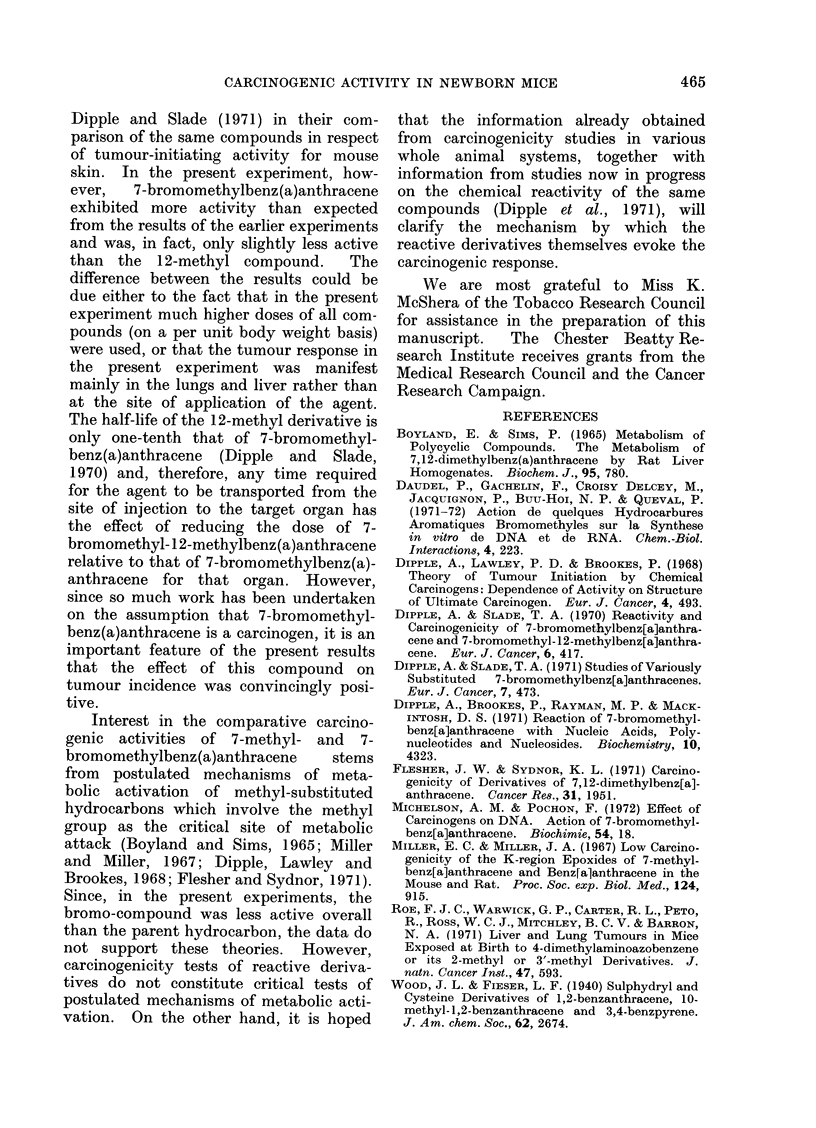# Carcinogenic Activity of Some Benz(a)Anthracene Derivatives in Newborn Mice

**DOI:** 10.1038/bjc.1972.63

**Published:** 1972-12

**Authors:** F. J. C. Roe, A. Dipple, B. C. V. Mitchley

## Abstract

Equimolar doses of 7-methylbenz(a)anthracene and 3 of its derivatives were given to newborn male and female Swiss mice. All 4 substances tested increased the risk of tumour development compared with that seen in control mice given the vehicle, arachis oil, only.

7-Methylbenz(a)anthracene itself was the most actively tumorigenic of the compounds studied, giving rise to subcutaneous sarcomata at the site of injection, and multiple lung tumours and liver tumours. 7-Bromomethyl-12-methylbenz(a)-anthracene was similarly active in the lung and liver but evoked fewer subcutaneous sarcomata. 7-Bromomethylbenz(a)anthracene was seemingly slightly less active than either 7-methylbenz(a)anthracene or 7-bromomethyl-12-methylbenz(a)anthracene. 4-Chloro-7-bromomethylbenz(a)anthracene exhibited only marginal activity in that it slightly increased the risk of liver tumour development in male mice.


*Br. J. Cancer* (1972) **26**, 461.

### CARCINOGENIC ACTIVITY OF SOME BENZ(a)ANTHRACENE DERIVATIVES IN NEWBORN MICE

F. J. C. ROE,* A. DIPPLE AND B. C. V. MITCHLEY

*From the Chester Beatty Research Institute, Institute of Cancer Research, Royal Cancer Hospital, Fulham Road, London, SW3 6JB*

Received 18 May 1972. Accepted 10 July 1972

**Summary.—Equimolar doses of 7-methylbenz(a)anthracene and 3 of its derivatives were given to newborn male and female Swiss mice. All 4 substances tested increased the risk of tumour development compared with that seen in control mice given the vehicle, arachis oil, only.**

**7-Methylbenz(a)anthracene itself was the most actively tumorigenic of the compounds studied, giving rise to subcutaneous sarcomata at the site of injection, and multiple lung tumours and liver tumours. 7-Bromomethyl-12-methylbenz(a)-anthracene was similarly active in the lung and liver but evoked fewer subcutaneous sarcomata. 7-Bromomethylbenz(a)anthracene was seemingly slightly less active than either 7-methylbenz(a)anthracene or 7-bromomethyl-12-methylbenz(a)anthracene. 4-Chloro-7-bromomethylbenz(a)anthracene exhibited only marginal activity in that it slightly increased the risk of liver tumour development in male mice.**

THE relation between structure and carcinogenic activity of a series of 7-bromomethylbenz(a)anthracenes has previously been investigated by the use of test systems in which the incidence of tumours at the site of a single application of the agent is the measure of activity. The use of such systems minimizes the risk of interference by factors such as peculiarities in transport of an agent to a distant target tissue (Dipple and Slade, 1970, 1971). Whilst these studies allowed the series of 7-bromomethylbenz(a)anthracenes to be arranged in order of carcinogenic potency, they did not provide a convincing demonstration of the carcinogenic activity of 7-bromomethylbenz(a)-anthracene itself, which compound is the most extensively studied member of this series (for example, Dipple *et al.*, 1971; Michelson and Pochon, 1972 and references therein; Daudel *et al.*, 1971–72).

We have studied the development of tumours after the administration of 7-bromomethylbenz(a)anthracene to newborn mice, a test system known to be sensitive to a wide variety of chemical carcinogens (Roe *et al.*, 1971 and references cited therein). The bromo-compounds that were most active, and least active in the previous tests, namely 7-bromomethyl-12-methylbenz(a)anthracene and 4-chloro-7-bromomethylbenz(a)-anthracene respectively, together with the parent hydrocarbon, 7-methylbenz(a)-anthracene, were included in the present study for comparison.

### MATERIALS AND METHODS

*Chemical agents.*—7-Bromomethylbenz(a)-anthracene, 7-bromomethyl-12-methylbenz-(a)anthracene and 4-chloro-7-bromomethyl-benz(a)anthracene were prepared as described previously (Dipple and Slade, 1970, 1971). 7-Methylbenz(a)anthracene was prepared by reduction of 7-bromomethyl-benz(a)anthracene with stannous chloride/HCl (Wood and Fieser, 1940).

* Present address: Tobacco Research Council, Glen House, Stag Place, London, S.W.1.

*Animal experiments.*—Litters from Swiss female mice obtained from a pathogen-free unit were grouped at random for treatment so that each group would consist of 70–80 survivors at the time of weaning.

Mice were given a single subcutaneous injection of test compound in 0·02 ml arachis oil on each of the first 3 days of life. Group A received 7-methylbenz(a)anthracene (200 μg per injection); Group B received 7-bromomethylbenz(a)anthracene (266 μg per injection); Group C received 4-chloro-7-bromomethylbenz(a)anthracene (294 μg per injection); Group D received 7-bromomethyl-12-methylbenz(a)anthracene (277 μg per injection) and Group E received arachis oil only. These doses are the molar equivalents of 200 μg of 7-methylbenz(a)anthracene.

Injections were made by introducing a fine-gauge needle through the skin near the root of the tail and threading it under the skin to deliver the injected material in the interscapular region.

After weaning at 3 weeks of age, males and females were caged separately (in groups of 5) in metal boxes containing wood shavings. The mice were fed on a standard diet (Formulation 86 from Messrs C. Holdman and Son (Plowco Feeds), Byers Lane, South Godstone, Surrey) and water was given *ad libitum.*

Mice were examined daily as to their general state of health, and more closely at weekly intervals for palpable tumours and other lesions. Mice with palpable tumours and any that were sick were killed and examined carefully by a standard post mortem procedure. The experiment was ended when the mice were between 57 and 61 weeks old. A full routine post mortem examination, which included distension of the urinary bladder with fixative, was carried out. Examination of the brain and spinal cord was not undertaken. The number of lesions thought to be neoplasms, and the sizes of the largest of these in each organ affected, were recorded.

All tissues with neoplasms or other lesions were examined histologically. Tissues were fixed in Bouin's solution and 5 μ paraffin wax sections were prepared and stained with haematoxylin and eosin.

### RESULTS

The results are summarized in Tables I and II.

*Survival.*—Between weaning and the termination of the experiment, at 401–431 days, more mice in Groups A and D died, or had to be killed because they were sick or had large tumours, than in the other groups. Post mortem examination was not possible in 22 mice because of decomposition (Group A: 1 ♂, Group B: 1 ♂, Group C: 1 ♀, Group D: 11 ♂ and 5 ♀ and Group E: 3♀).

The data recorded in Table I and in the following section of the text refer only to mice that were examined post mortem.

*Sarcomata at the site of injection* (see Table I).—Eleven mice of Group A, 1 of Group B and 2 of Group D developed sarcomata at the site of subcutaneous injection. Histologically all these tumours, except 2 of those in Group A, were fibrosarcomata. In one Group A female, the tumour was a pleomorphic sarcoma and in one male it was a rhabdomyosarcoma.

*Lung tumours* (see Table II).—No lung tumours were seen in 34 control males (Group E) but one of 40 control females developed a single small lung tumour. One of 33 males and one of 38 females in Group C each developed a solitary lung tumour. By contrast, a high incidence of lung tumours was seen in both the males and females of Groups A, B and D. In terms of incidence of mice with tumours, tumour multiplicity, and average size of the largest tumour, the response was slightly less marked in the females of Group B than in those of Groups A and D, whereas the response in males was somewhat greater in Group D than in Groups A and B.

The lung tumours ranged in histological appearance from non-invasive adenomata (Grade 1) through locally invasive adenocarcinomata (Grade 2) to adenocarcinomata showing metastases *via* the airways to other parts of the lobe of origin (Grade 3) and adenocarcinomata showing invasion of the chest wall (Grade 4). No case of metastasis to extrathoracic sites (Grade 5) was seen. The incidence of

TABLE I.—*Details of Treatment, Incidence of Malignant Lymphoma and Incidence of Sarcomata at the Site of Subcutaneous Injection*

| Group | Treatment (in 0·02 ml arachis oil on each of first 3 days of life) | No. of mice alive at 21 days that were subsequently examined post mortem | No. of mice that developed malignant lymphoma | Times of death of mice with lymphoma (days) | No. of mice that developed sarcoma at the site of injection | Times of death of mice with sarcoma (days) |
|---|---|---|---|---|---|---|
| A | 200 μg 7-Methylbenz(a)anthracene | ♂ 39 | 1 | 186 | 7 | 128, 187, 233, 245, 267, 281, 302 |
| | | ♀ 33 | 2 | 160, 235 | 4 | 181, 186, 188, 266 |
| B | 266 μg 7-Bromomethylbenz(a)anthracene | ♂ 43 | 0 | — | 0 | — |
| | | ♀ 28 | 2 | 324, 330 | 1 | 176 |
| C | 294 μg 4-Chloro-7-bromomethylbenz(a) anthracene | ♂ 33 | 0 | — | 0 | — |
| | | ♀ 38 | 0 | — | 0 | — |
| D | 277 μg 7-Bromomethyl-12-methylbenz(a) anthracene | ♂ 29 | 2 | 162, 379 | 2 | 162, 331 |
| | | ♀ 25 | 0 | — | 0 | — |
| E | Arachis oil only | ♂ 34 | 0 | — | 0 | — |
| | | ♀ 40 | 1 | 382 | 0 | — |

TABLE II.—*Incidence of Lung Tumours*

| Group | Sex | Mice with one or more lung tumours/Mice examined post mortem: Before 300 days | 301–400 days | 401–431 days | % of mice dying after 300 days with one or more lung tumours (% with multiple tumours): ♂ | ♀ | Mice with one or more liver tumours/Mice examined post mortem: Before 300 days | 301–400 days | 401–431 days | % of mice dying after 300 days with one or more liver tumours (% with multiple tumours): ♂ | ♀ |
|---|---|---|---|---|---|---|---|---|---|---|---|
| A | ♂ | 5/10 | 14/18 | 8/11 | 76 (72) | | 2/10 | 14/18 | 10/11 | 83 (83) | |
| | ♀ | 3/9 | 8/9 | 14/15 | | 92 (92) | 0/9 | 2/9 | 3/15 | | 21 (12) |
| B | ♂ | — | 9/11 | 24/32 | 77 (67) | | — | 8/11 | 27/32 | 81 (72) | |
| | ♀ | 0/2 | 5/12 | 10/14 | | 58 (46) | 0/2 | 0/12 | 1/14 | | 4 (4) |
| C | ♂ | 0/1 | — | 1/32 | 3 (0) | | 0/1 | — | 8/32 | 25 (9) | |
| | ♀ | — | 0/5 | 1/33 | | 3 (0) | — | 0/5 | 0/33 | | 0 (0) |
| D | ♂ | 1/3 | 21/23 | 3/3 | 92 (92) | | 1/3 | 20/23 | 3/3 | 88 (85) | |
| | ♀ | 3/8 | 11/12 | 5/5 | | 94 (94) | 0/8 | 0/12 | 3/5 | | 18 (0) |
| E | ♂ | 0/6 | — | 0/28 | 0 (0) | | 0/6 | — | 1/28 | 4 (0) | |
| | ♀ | 0/1 | 1/7 | 0/32 | | 3 (0) | 0/1 | 0/7 | 0/32 | | 0 (0) |

Grade 3 and Grade 4 lung tumours in the 5 groups was as follows:

Group A: 2 male and 3 female.
Group B: 0 male and 1 female.
Group C: 0 male and 1 female.
Group D: 2 male and 7 female.
Group E: 0 male and 0 female.

*Liver tumours* (see Table II).—One of 34 control males (Group E) developed a single liver tumour. Eight of 33 males of Group C developed liver tumours and in 3 of these multiple nodules were seen. In contrast, multiple liver tumours developed in the majority of males in Groups A, B and D. In terms of multiplicity of tumours and average size of the largest tumour, the response was similar in Groups A, B and D.

In females, no liver tumours were seen in the controls (Group E) nor in mice of Group C, but a low incidence was encountered in Groups A, B and D.

Histologically, all the liver tumours were derived from parenchymal cells. They varied in appearance from being difficult to distinguish from normal liver in cellular size and arrangement to being grossly abnormal in both these respects. Some were of a papillary arrangement and some showed evidence of local invasiveness, but none had invaded abdominal organs other than the liver and no distant metastases were seen.

*Malignant lymphoma.*—The only other neoplasms seen in the experiment were 8 cases of malignant lymphoma: 3 in Group A, 2 in Group B, 2 in Group D and 1 in Group E (Table I). In 2 cases the thymus was the organ principally involved (one female of Group A and one female of Group E). In all other instances there was generalized involvement of lymphatic tissues. Some of the tumours were of stem cell type and some of lymphocytic type.

*Other pathological changes.*—Foci of round cell infiltration were seen in the wall of the urinary bladder in a proportion of mice of all groups. The incidence of this change was not associated with treatment. No significant changes were observed in the bladder epithelium of any mice.

No changes of obvious significance were encountered in any other organ.

### DISCUSSION

Marked differences in tumour incidence were seen between the 4 groups. 7-Methylbenz(a)anthracene (Group A) was markedly more productive of sarcomata at the site of injection than any of the other compounds. 7-Methylbenz(a)anthracene (Group A), 7-bromomethylbenz-(a)anthracene (Group B) and 7-bromomethyl - 12 - methylbenz(a)anthracene (Group D) all evoked a high incidence of lung tumours in both male and female mice. 4-Chloro-7-bromomethylbenz(a)-anthracene (Group C), on the other hand, evoked no more lung tumours than did the vehicle only (Group E).

The sex difference in liver tumour incidence was not unexpected since male mice are generally more susceptible to the development of these tumours than females (Roe *et al.*, 1971). However, it is of interest that in the present experiment liver tumours tended to arise preferentially in the females of the groups in which the males were most severely affected.

The incidence of lymphoma was too low to provide an index of the relative tumorigenicity of the 4 compounds. The results with respect to the liver and lung tumours suggest that the 4 compounds can be ranked in the following descending order of tumorigenicity in the newborn mouse test system:

Most active
7-bromomethyl-12-methylbenz(a)-anthracene (Group D)
7-methylbenz(a)anthracene (Group A)
Less active
7-bromomethylbenz(a)anthracene (Group B)
Least active
4-chloro-7-bromomethylbenz(a)-anthracene (Group C)

This order is similar to that found by

Dipple and Slade (1971) in their comparison of the same compounds in respect of tumour-initiating activity for mouse skin. In the present experiment, however, 7-bromomethylbenz(a)anthracene exhibited more activity than expected from the results of the earlier experiments and was, in fact, only slightly less active than the 12-methyl compound. The difference between the results could be due either to the fact that in the present experiment much higher doses of all compounds (on a per unit body weight basis) were used, or that the tumour response in the present experiment was manifest mainly in the lungs and liver rather than at the site of application of the agent. The half-life of the 12-methyl derivative is only one-tenth that of 7-bromomethylbenz(a)anthracene (Dipple and Slade, 1970) and, therefore, any time required for the agent to be transported from the site of injection to the target organ has the effect of reducing the dose of 7-bromomethyl-12-methylbenz(a)anthracene relative to that of 7-bromomethylbenz(a)-anthracene for that organ. However, since so much work has been undertaken on the assumption that 7-bromomethylbenz(a)anthracene is a carcinogen, it is an important feature of the present results that the effect of this compound on tumour incidence was convincingly positive.

Interest in the comparative carcinogenic activities of 7-methyl- and 7-bromomethylbenz(a)anthracene stems from postulated mechanisms of metabolic activation of methyl-substituted hydrocarbons which involve the methyl group as the critical site of metabolic attack (Boyland and Sims, 1965; Miller and Miller, 1967; Dipple, Lawley and Brookes, 1968; Flesher and Sydnor, 1971). Since, in the present experiments, the bromo-compound was less active overall than the parent hydrocarbon, the data do not support these theories. However, carcinogenicity tests of reactive derivatives do not constitute critical tests of postulated mechanisms of metabolic activation. On the other hand, it is hoped that the information already obtained from carcinogenicity studies in various whole animal systems, together with information from studies now in progress on the chemical reactivity of the same compounds (Dipple *et al.*, 1971), will clarify the mechanism by which the reactive derivatives themselves evoke the carcinogenic response.

We are most grateful to Miss K. McShera of the Tobacco Research Council for assistance in the preparation of this manuscript. The Chester Beatty Research Institute receives grants from the Medical Research Council and the Cancer Research Campaign.

### REFERENCES

Boyland, E. & Sims, P. (1965) Metabolism of Polycyclic Compounds. The Metabolism of 7,12-dimethylbenz(a)anthracene by Rat Liver Homogenates. *Biochem. J.*, **95**, 780.

Daudel, P., Gachelin, F., Croisy Delcey, M., Jacquignon, P., Buu-Hoi, N. P. & Queval, P. (1971–72) Action de quelques Hydrocarbures Aromatiques Bromomethyles sur la Synthese *in vitro* de DNA et de RNA. *Chem.-Biol. Interactions*, **4**, 223.

Dipple, A., Lawley, P. D. & Brookes, P. (1968) Theory of Tumour Initiation by Chemical Carcinogens: Dependence of Activity on Structure of Ultimate Carcinogen. *Eur. J. Cancer*, **4**, 493.

Dipple, A. & Slade, T. A. (1970) Reactivity and Carcinogenicity of 7-bromomethylbenz[a]anthracene and 7-bromomethyl-12-methylbenz[a]anthracene. *Eur. J. Cancer*, **6**, 417.

Dipple, A. & Slade, T. A. (1971) Studies of Variously Substituted 7-bromomethylbenz[a]anthracenes. *Eur. J. Cancer*, **7**, 473.

Dipple, A., Brookes, P., Rayman, M. P. & Mackintosh, D. S. (1971) Reaction of 7-bromomethylbenz[a]anthracene with Nucleic Acids, Polynucleotides and Nucleosides. *Biochemistry*, **10**, 4323.

Flesher, J. W. & Sydnor, K. L. (1971) Carcinogenicity of Derivatives of 7,12-dimethylbenz[a]-anthracene. *Cancer Res.*, **31**, 1951.

Michelson, A. M. & Pochon, F. (1972) Effect of Carcinogens on DNA. Action of 7-bromomethylbenz[a]anthracene. *Biochimie*, **54**, 18.

Miller, E. C. & Miller, J. A. (1967) Low Carcinogenicity of the K-region Epoxides of 7-methylbenz[a]anthracene and Benz[a]anthracene in the Mouse and Rat. *Proc. Soc. exp. Biol. Med.*, **124**, 915.

Roe, F. J. C., Warwick, G. P., Carter, R. L., Peto, R., Ross, W. C. J., Mitchley, B. C. V. & Barron, N. A. (1971) Liver and Lung Tumours in Mice Exposed at Birth to 4-dimethylaminoazobenzene or its 2-methyl or 3′-methyl Derivatives. *J. natn. Cancer Inst.*, **47**, 593.

Wood, J. L. & Fieser, L. F. (1940) Sulphydryl and Cysteine Derivatives of 1,2-benzanthracene, 10-methyl-1,2-benzanthracene and 3,4-benzpyrene. *J. Am. chem. Soc.*, **62**, 2674.